# An investigation of the convergent validity and test–retest reliability of three uncertainty preference measures

**DOI:** 10.3758/s13428-025-02729-9

**Published:** 2025-07-09

**Authors:** Guangyu Zhu, Yiyun Shou, Michael Smithson

**Affiliations:** 1 School of Medicine and Psychology, The Australian National University, Canberra, ACT Australia; 2 Saw Swee Hock School of Public Health, National University of Singapore and National University Health System, Singapore, Singapore; 3 Lloyd’s Register Foundation Institute for the Public Understanding of Risk, National University of Singapore, Singapore, Singapore

**Keywords:** Reliability, Validity, Uncertainty preference, Measurement

## Abstract

Establishing a reliable and valid measure is crucial for ensuring the accuracy and replicability of research findings on risk and uncertainty preference. However, few studies have assessed the reliability and validity of behavioral measures of uncertainty preference. This study examined the convergent validity and test–retest reliability of three commonly used uncertainty preference measures: forced binary choice, certainty equivalent, and matching probability tasks. Experiments 1 (*N* = 302) and 2 (*N* = 366) tested the convergent validity and test–retest reliability of one-off assessment of these measures and found that the three measures did not demonstrate satisfactory convergent validity and test–retest reliability for the one-off assessment. Experiment 3 (*N* = 311) increased the number of repeats to explore whether repeated measurements could enhance convergent validity and test–retest reliability by addressing the attenuation effect of lack of reliability. The convergent validity between certainty equivalent and matching probability improved in the repeated measurement condition. However, the test–retest reliability of the three measures was still not satisfactory in repeated measurement conditions. These findings highlight the measurement issues in the behavioral measures of uncertainty preferences. The potential causes of this low validity and reliability of behavioral measures of uncertainty preference are discussed.

**Supplementary Information:**

The online version contains supplementary material available at 10.3758/s13428-025-02729-9.

## Introduction

Researchers from many disciplines, such as psychology and economics, are interested in people’s risk and uncertainty preference or attitudes. This interest motivates the development of various risk and uncertainty preference measures. Recently, there has been an increasing awareness of the importance of evaluating the reliability and validity of these measures used for dependent variables in experiments (Heukelom, 2011). However, the validity and reliability of the measures of risk and uncertainty preference seem to be controversial, especially for behavioral measures.

Some studies have evaluated the convergent reliability and test–retest validity of risk preference measures and have found that the behavioral risk preference measures lack good convergent reliability and test–retest validity, compared to questionnaire-based measures (Andersen et al., 2008; Beauchamp et al., 2017; Coppola, 2014; Crosetto & Filippin, 2016; Frey et al., 2017; Hey et al., 2009). This raises a concern about the findings based on these behavioral measures. Yet, few studies have explored the reliability and validity of behavioral measures of uncertainty preferences. It was found that the uncertainty preference revealed in some behavioral measures was inconsistent, and the findings about individuals’ uncertainty preference also varied across measures (Cavatorta & Schröder, 2019; Charness et al., 2013; Dimmock et al., 2016; Voorhoeve et al., 2016). This casts doubt on the reliability and validity of the behavioral measures of uncertainty preference.

While some studies have highlighted the unsatisfactory reliability and validity of behavioral measures of risk preference, few studies have systematically analyzed the underlying causes of this measurement issue. Therefore, this study aims to examine the validity and reliability of behavioral measures of uncertainty preference and explore potential causes and corresponding solutions to address their low validity and reliability.

### Risk and uncertainty preference measures

While various definitions exist, risk is most commonly defined as a situation with mathematically calculable probabilities, while uncertainty refers to a situation where the probabilities are unknown or cannot be accurately estimated (Knight, 1921). Uncertainty can be further divided into at least two subtypes: ambiguity and conflictive uncertainty. Ambiguity is defined as “a particular type of uncertainty due to the nature of one’s information concerning the relative likelihood of events” (Ellsberg, 1961, p. 657). It can be characterized by a probability interval describing a future uncertain outcome. Conflictive uncertainty is defined as “uncertainty arising from disagreement about states of reality that the cognizer believes cannot be true simultaneously” (Smithson, 2022, p. 2). It can be characterized by two conflicting probability estimations from equally credible sources when the conflictive uncertainty happens in probability. Ambiguity and conflictive uncertainty should be treated as two distinct types of uncertainty, which are associated with different brain regions (Pushkarskaya et al., 2010). Participants can also distinguish between the two types of uncertainty and have shown a preference for ambiguity over conflictive uncertainty (Cabantous, 2007; Cabantous et al., 2011; Smithson, 1999, 2015; Smithson et al., 2019).

The behavioral measures of risk and uncertainty preference include, but are not limited to, forced binary choice, certainty equivalent, and matching probability tasks. The forced binary choice (FBC) task presents participants with two options with different outcomes or different probabilities related to the outcomes, asking them to choose the one they prefer (Binmore et al., 2012; Kocher et al., 2018; Smithson, 1999; Smithson et al., 2019; Visschers, 2017). This task has been applied in many risk preference tasks, such as the multiple price list task (Holt & Laury, 2002; Chakravarty & Roy, 2008) and the adaptive lotteries task (Rieskamp, 2008). It is also frequently used in ambiguity and conflictive uncertainty preference studies, such as Fox and Tversky (1995) and Smithson (1999).

Certainty equivalent (CE) is an immediate guaranteed outcome that people would accept in exchange for a future uncertain outcome (Simon, 1956; Theil, 1957). The task of capturing CE usually provides participants with multiple pairwise comparisons and tries to find the point where they are indifferent between two options. One option is to win (lose) a certain amount of money, and the other option is a uncertain positive (negative) outcome. CE was first proposed as a measure of risk preference (or so-called risk premium) but is also applied to measure ambiguity preference (Güney & Newell, 2019; Krahnen, 1997; Smithson & Campbell, 2009).

Matching probability (MP) is the precise probability that people will accept in exchange for an uncertain probability. This was developed by Dimmock et al. (2016) as a measure of ambiguity preference and has been used in two studies on ambiguity aversion (Baillon et al., 2018a, b). The procedure for MP tasks is similar to that of CE tasks, aiming to find the point at which individuals are indifferent between options. However, one option in the MP task is with a precise probability and the other is with an uncertain probability (either interval or conflicting). When the individuals are indifferent between options, the precise probability will be the matching probability for the uncertain probability.

### Convergent validity and test–retest reliability of behavioral measures in risk and uncertainty preference

The current reliability and validity tests of measures of risk and uncertainty preference have primarily focused on their convergent validity and test–retest reliability. These criteria are addressed first because they ensure that the measures can consistently (test–retest reliability) and accurately (convergent validity) capture the defined construct (Beauchamp et al., 2017; Frey et al., 2017).

Convergent validity assesses the extent to which the measures are capturing the same construct. According to Gregory (2004), measures claiming to capture the same construct should have a correlation of at least 0.5 to achieve acceptable convergent validity. Test–retest reliability evaluates whether the results from the measure are consistent over time. It is usually assessed by the correlation of individuals’ scores between two measurement times (usually 7–14 days). A correlation of 0.8 could be regarded as evidence supporting the test–retest reliability of the measures (Mohajan, 2017).

Previous research in risk and ambiguity preferences typically assumes monotonicity and procedural or measurement invariance (Apesteguia & Ballester, 2018; Kelsey & Quiggin, 1994; Millner et al., 2013; Tversky & Kahneman, 1992). These assumptions imply that different measures of uncertainty preferences should exhibit high reliability (e.g., internal consistency and test–retest reliability) and convergent validity. The monotonicity assumption holds that individuals should consistently prefer options with higher expected utility. Procedural or measurement invariance requires such preferences to be stable regardless of the elicitation procedure (e.g., framing, timing, or the measurement method) used. Under these assumptions, an individual who reports a higher CE or MP for one option over another—indicating a preference for the former—should also be expected to choose that option in an FBC task. Such preference should also remain stable across different contexts and over time. Moreover, when both CE and MP tasks are used to capture the preference of an option, their results should be directly associated with the subjective expected utility of the option (Friedman & Savage, 1952). Therefore, a high degree of convergent validity is expected between the two tasks when measuring uncertainty preferences.

However, previous research has shown that commonly used behavioral measures of risk and uncertainty preferences often lack strong convergent validity and test–retest reliability, diverging from these theoretical expectations (Andersen et al., 2008; Frey et al., 2017; Crosetto & Filippin, 2016). For example, Frey and colleagues (2017) examined the convergent validity among eight behavioral risk preference tasks and 22 questionnaire-based measures of risk propensity. They found that correlations among the behavioral measures were weak (*r* < 0.10), and their correlations with the questionnaire-based measures were even lower (*r* < 0.06). Similarly, Crosetto & Filippin (2016) reported that behavioral measures of risk preference were only weakly correlated with self-reported risk propensity measures (*r* < 0.30).

Low convergent validity of these behavioral measures has been consistently observed across a wide range of tasks and instruments, raising questions about whether these measures reflect a unitary, stable underlying trait. Berg et al. (2005) found no significant correlations among participants’ rankings across three commonly used behavioral tasks, including the Becker–DeGroot–Marschak task, the English Clock Auction, and the First-Price Auction. Galizzi and Miniaci (2016) found low correlations between two behavioral risk attitude tasks, the Holt–Laury (HL) and Eckel–Grossman (B-EG), ranging from *r* = 0.16 to 0.17. Their correlations with the Socio-Economic Panel (SOEP) general risk item were even lower, ranging from *r* = 0.09 to 0.14. Grüner et al. (2023) also found substantial divergence across elicitation methods of risk attitude: when comparing HL, EG, and the SOEP item, only 55.2–56.3% of participants were classified into the same risk categories (risk-averse, risk-neutral, or risk-seeking) across any two measures, underscoring the limited convergence among methods. As for reasons, Berg et al. (2005) argued that such divergence may result from the different cognitive frames invoked by these tasks, such as valuation versus competition. Galizzi and Miniaci (2016) and Grüner et al. (2023) suggested that it may also reflect differences in the interpretation of “risk,” with self-report items capturing perceptions of real-world recklessness, while behavioral tasks focus more narrowly on outcome variance.

Measures of ambiguity preference, meanwhile, have demonstrated similar, and in some cases greater, instability than those of risk preference. Duersch et al. (2017) found that only 57% of participants maintained consistent ambiguity preferences over a 2-month interval, compared to 85% consistency for risk preferences during the same period. Similarly, Xu et al. (2024) examined ambiguity and risk attitudes across both monetary and medical domains, reporting moderate cross-domain correlations for risk preferences (*r* = 0.48–0.54) and slightly lower correlations for ambiguity preferences (*r* = 0.34–0.42). They also found that ambiguity preferences exhibited weaker test–retest reliability (*r* = 0.29–0.32) than risk preferences (*r* = 0.46–0.61). Duersch et al. (2017) attributed this instability to participants’ inability to recall their earlier decisions, suggesting that risk and ambiguity preferences may reflect memory-dependent responses. In contrast, Xu et al. (2024) emphasized the influence of emotional and contextual factors. Ambiguity preferences may be more unstable because the brain regions involved in processing ambiguity, such as the orbitofrontal cortex, are more sensitive to transient emotional states (Hsu et al., 2005). These findings together indicate that ambiguity preferences elicited through behavioral tasks may suffer from even greater measurement issues than risk preference measures.

### Potential factors contributing to the low convergent validity

In addition to the reasons mentioned above, the low convergent validity among behavioral measures may be attributed to several other factors. First, the different sources of systematic errors can lead to low convergent validity. Behavioral measures often capture additional systematic errors beyond the defined construct, and these systematic errors may not be shared among other measures, resulting in low convergent validity. Removing systematic errors from the measured scores is unrealistic, given that they are inherent to the measures. However, the evaluation of the convergent validity should take all possible sources of variance into consideration (Bishop & Boyle, 2019). For example, in the case of uncertainty preference measures, CE tasks can mix participants’ ambiguity preference (preference for precise probability) with their risk preference (preference for certainty in outcome), while MP tasks do not (Theorem 3.1; Dimmock et al., 2016). This distinction may potentially lead to low convergent validity between CE and MP, as the two measures have different sources of error.

Second, the low convergent validity among behavioral measures could be attributed to the low reliability of these measures. Frey et al. (2017) found that the 6-month test–retest correlations of eight behavioral measures varied from 0.29 to 0.63. Similarly, Lönnqvist et al. (2015) found that gambling-based risk preference measures had a rank correlation of only 0.26. The evaluation of convergent validity can be attenuated by the unreliability of the measures (Cochran, 1968). For example, given two variables, ${X}{\prime}$ and ${Y}{\prime}$, measured as $X$ and $Y,$ with correlation ${r}_{XY}$ and the reliability of each as ${r}_{XX}$ and ${r}_{YY}$, the true correlations between them would be

$${r}_{{X}{\prime}{Y}{\prime}}=\frac{ {r}_{XY}}{\sqrt{{r}_{XX}{r}_{YY}}}$$

Suppose there are two measures, each with a reliability of 0.5. The correlation between them, typically viewed as an estimation of the convergent validity, would only capture half of their true underlying correlation.

There are two ways to address this low reliability. Reliability is the ratio between true score variance and total variance (Lord & Novick, 2008). The low test–retest reliability findings suggest that measurement error, particularly random error, constitutes a large proportion of the total variance. Thus, to increase the reliability of these measures, experimenters can increase measurement repetitions and average responses from multiple repetitions to balance the random error in the measurement process. This can effectively reduce the proportion of measurement errors and enhance the reliability of the measures, which could ultimately improve the convergent validity between measures.

Alternatively, averaging preferences across a sample may help balance out random error at the individual level, resulting in more stable and consistent scores over time. To assess this consistency, researchers can perform an agreement test, often operationalized through a paired *t*-test (Aldridge et al., 2017; Berchtold, 2016). In such analyses, it is expected that the difference in participants’ scores across two measurement occasions should not significantly deviate from zero. Moreover, these differences should fall within an acceptable range, indicating that the measure can reliably capture average preferences over time. This type of evidence can support the use of risk and uncertainty preference measures in studies focused on group-level trends or population averages, even when individual-level reliability is low.

Moreover, contextual factors can influence respondents’ decision-making behavior, potentially affecting the convergent validity and test–retest reliability of different measures. For example, people’s preferences are often constructed in response to contextual cues, such as the domain of the decision (Peters, 2006; Mukerji & Tallon, 2001; Weber et al., 2002; Warren et al., 2010). As a result, measures can exhibit differing levels of validity and reliability depending on the scenarios. Decisions in a gambling scenario may be treated as a monetary calculation or a matter of “luck,” whereas decisions in health or medical domains (e.g., treating patients or personal health risks) can involve high-stakes consequences and moral considerations (Draper, 2001). The high stakes of health and medical decisions may prompt participants to engage more thoughtfully, potentially reducing random noise in their responses.

Additionally, the framing of a decision can influence which cognitive and emotional processes become salient in shaping preferences (Bier & Connell, 1994; Voorhoeve et al., 2016). In gain-framed scenarios, risk preferences are motivated by a conservative, certainty-seeking mindset, whereas in loss-framed scenarios, individuals tend to become risk-seeking, driven by a strong desire to avoid losses (Tversky & Kahneman, 1992). Similarly, ambiguity preferences vary across different frames: individuals may display ambiguity-aversion behavior in gain-framed scenarios, to avoid the potential lower bound of the winning chance, but prefer more ambiguity in loss-framed scenarios, to chase a lower likelihood of loss (Kocher et al., 2018). As such, convergent validity among behavioral measures under different frames may reflect the different cognitive and emotional processes underlying risk or uncertainty preferences. Although these measures are often assumed to assess the same constructs, it is essential to investigate convergent validity and test–retest reliability across different contexts and frames to fully understand their psychometric property.

### The current study

Although various behavioral tasks have been used to assess individual differences in uncertainty preference, knowledge of their psychometric properties is still lacking. This study presents a psychometric evaluation of three behavioral measures (forced binary choice, certainty equivalent, and matching probability) in assessing preferences for two types of uncertainty: ambiguity and conflictive uncertainty. The study incorporates relevant variations in decision framing (gain vs. loss) and scenario type (gambling vs. medical).

In this study, traditional significance testing in correlation analyses is supplemented with Bayes factors and confidence intervals, to address three research questions:

1. Do preferences measured by different measures yield significantly positive correlations? (assessed via significance test)
2. Are these correlations strong enough to support psychometric expectations? (assessed via Bayes factors)
3. If not, how strong are the associations? (assessed via confidence intervals)

Research on decision-making under uncertainty often relies on assumptions of monotonicity and procedural invariance (e.g., Tversky & Kahneman, 1992; Apesteguia & Ballester, 2018). These assumptions imply that if individuals have stable and well-ordered preferences, those preferences should manifest consistently across measurements and over time, yielding a strong convergent validity (conventionally *r* > 0.5, Gregory, 2004) and test–retest reliability (conventionally *r* > 0.8, Mohajan, 2017). To evaluate the psychometric adequacy of these measures, hypotheses were specified predicting that both their convergent validity and test–retest reliability would exceed acceptable thresholds. Although these hypotheses are not supported by prior empirical findings, testing them directly using Bayes factors allows for a more precise quantification of the strength of evidence against them, an aspect largely overlooked in previous studies. This approach enables a more informative rejection of presumed theoretical expectations and underscores the extent to which current behavioral measures may fall short of established psychometric standards. Based on these theoretical expectations, this study proposes two hypotheses:

- Hypothesis 1: The convergent validity among preferences elicited by the three measures will exceed an acceptable threshold regardless of uncertainty types, decision frames, and scenario types (*r* >0.5).
- Hypothesis 2: The test–retest reliability of each measure will exceed an acceptable threshold across measurement occasions, regardless of uncertainty types, decision frames, and scenario types (*r* >0.8).

Three experiments were conducted to evaluate these hypotheses, with a focus on identifying potential causes if low convergent validity were to be be observed: Experiment 1 assessed the convergent validity of the three behavioral tasks across both gain and loss frames, and explored whether systematic sources of error may explain observed low convergent validity. Experiment 2 extended the evaluation to include test–retest reliability across two time points, investigating whether low reliability accounts for observed low convergent validity. Experiment 3 introduced a medical decision-making context and increased the number of repeated measurements to examine whether increasing the number of measurement trials could improve both test–retest reliability and convergent validity.

## Experiment 1

### Method

#### Participants and design

A total of 302 English-speaking adults from the USA and UK were recruited from the Prolific platform and participated in this experiment, with 171 of them allocated to the gain domain and 131 to the loss domain. The experiments in the gain and loss domains were conducted separately. According to G*Power (Faul et al., 2007), these sample sizes are sufficient to detect a medium-sized correlation (*r = *0.3) at the 0.05 significance level, with statistical power of 0.99 and 0.97, respectively. The participants were compensated at a fixed rate of £0.8 for their participation in a survey with no additional incentive, for which the median duration was expected to be 7 minutes.

The mean participant age was 36.04 (*SD* = 13.50) years; 48% were male, 47% were female, and 5% non-binary gender. The participants included 75% Caucasian, 6% with African heritage, 12% Asian, and 7% with other racial backgrounds. There were no significant differences in these demographic variables between the gain and loss domains.

This experiment used a mixed design, with type of uncertainty as a within-subject variable and type of domain as a between-subject variable. Each participant completed the four tasks sequentially, measuring their risk and uncertainty preferences.

#### Materials

Participants completed the forced binary choice tasks, certainty equivalent tasks, and matching probability tasks in randomized order. In each task, they were required to choose from different gambling options, each offering varying winning probabilities and amount of money.

##### Forced binary choice task

In the forced binary choice (FBC) task, participants were required to indicate their preference between two options (see Figure A1 in Appendix A for details). Option 1 is an example of conflictive uncertainty, where two forecasters provided precise but conflicting estimations of the probability of winning or losing money. Option 2 is an example of ambiguity, with two forecasters providing identical but imprecise estimations of the probability of winning or losing money. The content of the two options was derived from previous studies on ambiguity and conflictive uncertainty preference (Cabantous, 2007; Cabantous et al., 2011; Güney & Newell, 2019; Smithson, 1999; Smithson et al., 2019).

**Fig. 1:**
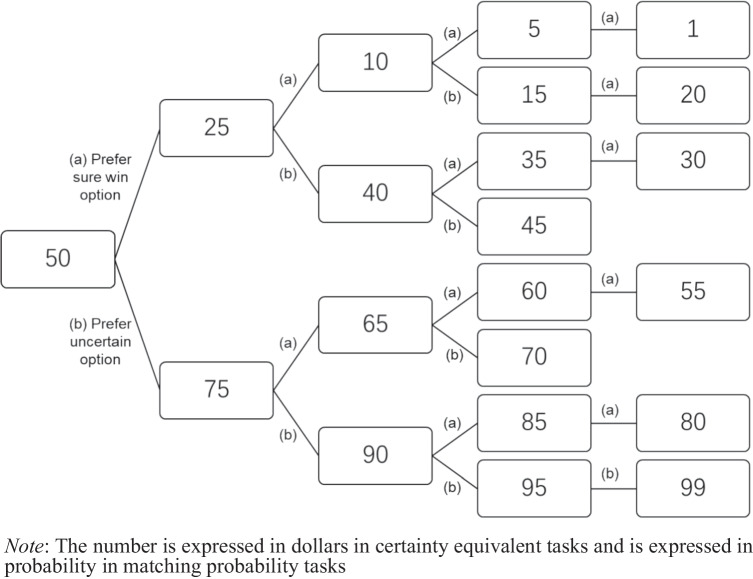
The procedure for the multiple pairwise comparisons

##### Certainty equivalent task

In the certainty equivalent (CE) task, participants were required to indicate their preference in multiple pairwise comparisons (see Figure A2 in Appendix A for details). Option 1 represented an uncertain option, which could be either ambiguity or conflictive uncertainty, while Option 2 was a sure-win/sure-loss option. In the gain domain, the amount of money in the sure-win option varied from $0 to $100 across the comparisons. Participants were presented with three options in each comparison: “Prefer Option 1,” “Prefer Option 2,” or “No preference.” The amount of money in the sure-win option for the first paired comparison was $50. If a participant preferred the sure-win option, they were presented with a second pair where the amount of money in the sure-win option decreased to $25 (the midpoint between $50 and $0). On the other hand, if they preferred the uncertain option, the amount of money in the sure-win option increased to $75 (the midpoint between $100 and $50). This procedure (as shown in Fig. 1) continued until participants either showed no preference between the two options or switched their preference (e.g., preferring the sure-win option when the sure-win was $45 but preferring the ambiguity option when the sure-win was $40). If participants showed no preference between the two options, then the amount of money in the sure-win option was treated as the certainty equivalent (CE) of the uncertain option. If they switched their preference within a $5 interval, then the midpoint of the interval was considered the CE of the uncertain option (e.g., $47.5 in the example provided). The procedure in the loss domain was similar, except that the sure-win option was changed to a sure-loss option.

##### Matching probability task

In a matching probability (MP) task, participants were also required to indicate their preferences in multiple pairwise comparisons (see Figure A3 in Appendix A for details). The procedure was similar to the CE task. The difference is that the sure-win/sure-loss option was replaced by an option with a precise probability of winning or losing $100. The probability of winning or losing $100 in the precise probability options varied from 0% to 100% across comparisons.

##### Risk preference task

Participants’ risk preference in the gain domain was assessed using the list of paired lotteries proposed by Holt and Laury (2002). Participants were presented with 10 ordered choices between two lotteries denoted as A or B (see Table B1 in Appendix B for details). Lottery A always paid either $100 or $80, while Lottery B paid $190 or $5. The probability that both lotteries paid the high payoff varied between choices from 10% to 90%.

Lottery A was considered safer than Lottery B. However, the expected value of Lottery A increased from $82 to $100, while the expected value of Lottery B increased from $23.5 to $190. For the first four choices, only risk-seeking subjects should choose Lottery B, as this lottery had a lower expected value, and more risk compared to Lottery A. After these choices, risk-averse subjects might switch to Lottery B. The later they switched to Lottery B, the more risk-averse they were.

Participants’ risk preference in the loss domain was assessed using a similar list of paired lotteries proposed by Chakravarty and Roy (2008). Their risk preference task, similar to Holt and Laury’s (2002), involved presenting participants with 10 ordered choices between two options labeled A and B. The payoff matrix of Chakravarty and Roy’s (2008) task is slightly different from Holt and Laury’s (2002) task (see Table B2 in Appendix B).

#### Procedure

Participants were invited to an online study hosted on the Qualtrics survey platform. The survey included three uncertainty preference tasks (the certainty equivalent, matching probability, and forced binary choice tasks) and one risk preference task. The order of the uncertainty preference tasks and the risk preference task was randomized, with half of the participants starting with the uncertainty preference tasks and the other half beginning with the risk preference task.

In the uncertainty preference tasks, participants were first provided with an introduction to the different types of options they would encounter in the formal tasks and completed practice sessions for each task variant. Subsequently, they completed the formal tasks. The order of the tasks was randomized. Upon completion of all tasks, participants completed the demographic questions asking their gender, age, race, education level and English proficiency.

### Results

The means, medians, and standard deviations of participants’ scores in the CE and MP tasks for ambiguity and conflictive uncertainty, and the proportion of participants preferring each kind of uncertainty in the FBC task are summarized in Table 1.

**Table 1:** Descriptive information for three ambiguity preference measures

	Ambiguity			Conflictive uncertainty		
Measure	Mean	Median	*SD*	Mean	Median	*SD*
Gain domain						
CE	30.56	32.50	17.59	31.04	32.50	18.45
MP	45.39%	47.50%	10.23	45.13%	47.50%	9.34
FBC	0.69			0.31		
Loss domain						
CE	45.01	50.00	21.92	43.48	50.00	22.53
MP	48.02%	50.00%	10.15	48.45%	50.00%	10.58
FBC	0.58			0.42		

CE = certainty equivalent; MP = matching probability; FBC = forced binary choice

#### Gain domain

The convergent validity between tasks was measured by both the Pearson’s correlation and Bayesian correlation (bayesfactor package: Morey et al., 2015). In Pearson’s correlation analysis, the null hypothesis H_0_ is *r* = 0 and the alternative hypothesis H_1_ is *r* $\ne$ 0. The *p* value and 95% confidence interval were calculated. In the Bayesian correlation analysis, the null hypothesis H_0_ is *r* < 0.5, and the alternative hypothesis H_1_ is *r* $\ge$ 0.5. The prior distribution for correlation $\rho$ is a uniform distribution in the range [−1, 1], so that all values within this range have equal prior likelihood. The Bayes factor for the alternative hypothesis BF_10_ was calculated to assess the likelihood that the correlation was above the acceptable criterion of good convergent validity (see Appendix C for details). The interpretation of BF_10_ is in accordance with the commonly used thresholds to define significance of evidence (Jeffreys, 1961).

To calculate the correlations between the FBC task and the other two tasks, the difference in scores between ambiguity and conflictive uncertainty in the CE (or MP) tasks was first calculated and then correlated with the binary choice from the FBC task. It was found that the correlation between the FBC and CE tasks was lower than 0.5, with *r* =.06, BF_10_ <0.001. Similarly, the correlation between the FBC and MP tasks was also lower than 0.5, with *r* = 0.20, BF_10_ <0.001. These Bayes factors showed extreme evidence for the null hypothesis (*r* < 0.5).

The correlations between the CE and MP tasks were calculated using the scores in the ambiguity or conflictive uncertainty condition separately. The correlation between the CE and MP tasks was lower than 0.5 both under ambiguity, *r* =.08, BF_10_ <0.001, and under conflictive uncertainty, *r* =.16, BF_10_ <0.001. These Bayes factors showed extreme evidence for the null hypothesis (*r* < 0.5). All the correlations were below the criterion of acceptable convergent validity, failing to support Hypothesis 1 (see Table 2).

**Table 2:** Convergent validity between different ambiguity preference measures

	*r*	95% CI	*p* (*r* = 0)	BF_10_ (*r* $\ge$ 0.5)
CE vs MP – A	.08	[−.07,.23]	.301	<0.001
CE vs MP – C	.16	[.07,.30]	.039	<0.001
FBC vs CE	.06	[−.09,.21]	.424	<0.001
FBC vs MP	.20	[.06,.34]	.007	<0.001

CE = certainty equivalent; MP = matching probability; FBC = forced binary choice; A = ambiguity; C = conflictive uncertainty

The relationship between the CE/MP tasks and the risk preference task was also assessed by both the Pearson’s correlation and Bayesian correlation analysis. In both analyses, the null hypothesis H_0_ is* r* = 0, and the alternative hypothesis H_1_ is* r* $\ne$ 0.

The correlations between the CE and risk preference tasks were significantly different from zero under both kinds of uncertainty (ambiguity: *r* =.35, BF_10_ > 100; conflictive uncertainty, *r* =.31, BF_10_ > 100). The Bayes factors showed extreme evidence for the alternative hypothesis (*r* $\ne$ 0). In contrast, the correlations between the MP and risk preference tasks were not significantly different from zero under either kind of uncertainty, ambiguity, *r* = −.01, BF_10_ = 0.180; conflictive uncertainty, *r* =.06, BF_10_ = 0.127. The Bayes factors showed moderate evidence for the null hypothesis (*r* = 0). These findings indicate that the uncertainty preference measured by the CE task has an overlap with participants’ risk preferences in gain domain, but the uncertainty preference measured by the MP tasks is not associated with participants’ risk preferences (see Table 3).

**Table 3:** Correlation between different ambiguity preference measures and the risk preference measure

	*r*	95% CI	*p* (*r* = 0)	BF_10_ (*r* $\ne$ 0)
CE – A	.35	[.21,.48]	<.001	>100
CE – C	.31	[.17,.44]	<.001	>100
MP – A	−.01	[−.16,.14]	.854	0.180
MP – C	.06	[−.09,.21]	.452	0.127

CE = certainty equivalent; MP = matching probability; FBC = forced binary choice; A = ambiguity; C = conflictive uncertainty

#### Loss domain

The analysis in the loss domain is the same as the one in the gain domain. The correlation between the FBC and CE tasks was lower than 0.5, *r* = −.05, BF_10_ < 0.001. Similarly, the correlation between the FBC and MP tasks was lower than 0.5, *r* =.02, BF_10_ < 0.001. The correlations between the CE and MP tasks were also lower than 0.5 under both kinds of uncertainty (ambiguity: *r* =.19, BF_10_ < 0.001; conflictive uncertainty: *r* =.29, BF_10_ < 0.001). These Bayes factors showed extreme evidence for the null hypothesis (*r* < 0.5). The correlations are all below the criteria of acceptable convergent validity, failing to support Hypothesis 1 (see Table 4).

**Table 4:** Convergent validity between different ambiguity preference measures

	*r*	95% CI	*p* (*r* = 0)	BF_10_ (*r* $\ge$ 0.5)
CE vs. MP – A	.20	[.03,.36]	<.001	<0.001
CE vs. MP – C	.29	[.14,.45]	.021	<0.001
FBC vs. CE	−.06	[−.23,.11]	.511	<0.001
FBC vs. MP	.03	[−.14,.20]	.766	<0.001

CE = certainty equivalent; MP = matching probability; FBC = forced binary choice; A = ambiguity; C = conflictive uncertainty

In terms of the relationship of CE/MP tasks with the risk preference task, the correlations between the CE and risk preference tasks were not significantly different from zero under either kind of uncertainty (ambiguity: *r* =.08, BF_10_ = 0.177; conflictive uncertainty: *r = *.14, BF_10_ = 0.449). The Bayes factors showed anecdotal to moderate evidence for the null hypothesis (*r = *0). The correlation between the MP and risk preference tasks was not significantly different from zero under ambiguity,* r* =.13, BF_10_ = 0.359. The Bayes factors showed anecdotal evidence for the null hypothesis (*r = *0). Similarly, the correlation between the MP and risk preference tasks was not significantly different from zero under conflictive uncertainty, *r = *.06, BF_10_ = 0.138. The Bayes factors showed moderate evidence for the null hypothesis (*r* = 0). These results indicate that neither of the uncertainty preferences revealed in the CE and MP tasks was associated with the risk preference task in the loss domain (see Table 5).

**Table 5:** Correlation between different ambiguity preference measures and the risk preference measure

	*r*	95% CI	*p* (*r* = 0)	BF_10_ (*r* $\ne$ 0)
CE – A	.08	[−.09,.25]	.323	0.177
CE – C	.15	[−.02,.31]	.090	0.449
MP – A	.14	[−.04,.30]	.121	0.359
MP – C	.06	[−.11,.23]	.489	0.138

CE = certainty equivalent; MP = matching probability; FBC = forced binary choice; A = ambiguity; C = conflictive uncertainty

#### Post hoc comparison between gain and loss domains

To compare the difference in results between the gain and loss domains, we used a *z*-test on Fisher z-transformed correlation coefficients. It demonstrated that there was no significant difference in the convergent validity between the gain and loss domains. The correlations between the FBC and CE tasks were not significantly different between the gain and loss domains (*z* = −1.02, *p* =.306). The correlations between the FBC and MP tasks were also not significantly different between the gain and loss domains (*z* = −1.47, *p* =.141). Similarly, the correlations between the CE and MP tasks were not significantly different between the two domains, under either ambiguity (*z* = 1.04, *p* =.296) or conflictive uncertainty (*z* = 1.26, *p* =.207).

However, there was a significant difference in the correlation between the CE/MP tasks and the risk preference tasks between the gain and loss domains. The CE task had a significantly higher correlation with the risk preference task in the gain domain than in the loss domain under ambiguity (*z* = 2.43, *p* =.015). In contrast, there were no significant differences in the correlations between the CE and risk preference tasks between the gain and loss domains under conflictive uncertainty (*z* = 1.44, *p* =.149). Similarly, there were no significant differences in the correlations between the MP and risk preference tasks between the gain and loss domains under either ambiguity (*z* = 1.28, *p* =.198) or conflictive uncertainty (*z* < 0.005, *p* >.995).

To further investigate whether the additional systematic error in the CE task (attributed to the association with risk preference) in the gain domain could contribute to low convergent validity between the CE and MP tasks, the partial correlations (ppcor package; Kim, 2015) between the CE and MP tasks in the gain domain were calculated after controlling for their association with the risk preference tasks. The partial correlation between the CE and MP tasks remained nonsignificant under both kinds of uncertainty after controlling for the additional systematic error associated with risk preference (ambiguity: *r*(169) =.09, *p* =.241; conflictive uncertainty: *r*(169) =.15, *p* =.055).

### Discussion

Experiment 1 demonstrated that the convergent validity among the three behavioral uncertainty preference measures fell below the acceptable criteria (*r* > 0.5; Gregory, 2004), suggesting that the elicited uncertainty preferences were inconsistent among the three measures. Additionally, it was observed that the CE tasks were correlated with the risk preference tasks in the gain domain, but not in the loss domain. This result suggests that the CE task assesses participants’ uncertainty preference in a way that is linked to their risk preference when the outcomes are about potential gain, which is consistent with Dimmock et al. (2016). However, the results of the partial correlation analysis ruled out the possibility that unshared systematic errors related to risk preference account for the low convergent validity between the CE and MP tasks.

## Experiment 2

Experiment 2 explored the low test–retest reliability of the uncertainty preference measures as a reason for the low convergent validity. Moreover, the difference in the correlations of the CE tasks and the risk preference tasks between the gain and loss domains in Experiment 1 might be attributed to the different risk preference tasks employed in the loss and gain domains. Therefore, this experiment tested the correlations between CE and the risk preference tasks in the gain domain, using a gain domain version of Chakravarty and Roy’s (2008) method.

### Method

#### Participants and design

A total of 366 participants, who were English speakers aged 18 and older from the USA and UK recruited via Prolific, participated in this experiment. This sample size is sufficient to detect a significant medium correlation (*r* = 0.3) with a 0.05 significance level and 0.99 power.

The mean age among participants was 36.87 (*SD* = 14.09) years, with 57% male, 39% female, and 4% non-binary genders. The participants included 7% African, 7% Asian, 77% Caucasian, and 9% people from other cultural backgrounds. About 73% of the participants (*n* = 268) completed the second measurement session 9 days after the first session. The remaining participants had a mean age of 37.83 (*SD* = 14.19), with 55% male, 41% female, and 4% non-binary genders. The participants included 7% African, 7% Asian, 79% Caucasian, and 7% people from other cultural backgrounds.

Participants were recruited through the online platform Prolific. They were compensated at a fixed rate of £0.8 for their participation in a survey with no additional incentive, for which the median duration was expected to be 7 minutes. This experiment was a within-subject design, and each participant completed all decision-making tasks.

#### Materials

The descriptions of the forced binary choice tasks, certainty equivalent tasks, and matching probability tasks are the same as in Experiment 1. Participants’ risk preference was measured using the list of paired lotteries developed by Chakravarty and Roy (2008).

#### Procedure

The procedure for the survey in each wave of data collection was the same as in Experiment 1. The invitation for the second wave was sent out 9 days after participants joined the first wave, and data collection for the second wave stopped after 14 days from their initial participation. Most of the participants (86%) joined the second wave on the day the invitation was sent out. Participants completed the risk preference task and demographic questions only once during the first wave of data collection. Participants completed the risk preference task only once as the baseline of their risk preference.

### Results

The means, medians, and standard deviations of participants’ scores in the CE and MP tasks for ambiguity and conflictive uncertainty, and the number of people who preferred different kinds of uncertainty in the FBC tasks are summarized in Table 6.

**Table 6:** Descrip information on three ambiguity preference measures

	Ambiguity					Conflictive uncertainty		
Measure	Mean	Median			*SD*	Mean	Median	*SD*
Time 1								
CE	28.72		26.25	18.47		27.27	27.50	18.10
MP	45.00%		47.50%	9.39		44.27%	47.50%	9.10
FBC	0.68					0.32		
Time 2 (9 days later)								
CE	29.06		27.50	16.55		29.94	27.50	18.19
MP	44.26%		47.50%	9.41		44.25%	47.50%	9.15
FBC	0.72					0.28		

CE = certainty equivalent; MP = matching probability; FBC = forced binary choice

The convergent validity among different uncertainty preference measures was calculated following the same procedure as in Experiment 1 (see Table 7). Similar to Experiment 1, the correlations between any two of the three tasks remained below 0.5 at both Time 1 and Time 2, with BF_10_ <0.001. These Bayes factors showed extreme evidence for the null hypothesis (*r* < 0.5). This suggests that their convergent validity is below the acceptable criteria, failing to support Hypothesis 1.

**Table 7:** Convergent validity between different preference measures

	Time 1				Time 2			
	*r*	95% CI	*p*	BF_10_	*r*	95% CI	*p*	BF_10_
			(*r* = 0)	(*r* $\ge$ 0.5)			(*r* = 0)	(*r* $\ge$ 0.5)
CE vs. MP – A	.17	[.07,.27]	<.001	<0.001	.15	[.03,.27]	.012	<0.001
CE vs. MP – A	.05	[−.05,.15]	.362	<0.001	.16	[.04,.27]	.010	<0.001
FBC vs. CE	.10	[−.00, 20]	.062	<0.001	.22	[.11,.34]	<.001	<0.001
FBC vs. MP	.24	[.14,.34]	<.001	<0.001	.03	[−.08,.15]	.557	<0.001

CE = certainty equivalent; MP = matching probability; FBC = forced binary choice; A = ambiguity; C = conflictive uncertainty

The test–retest reliability was assessed by the Pearson’s correlation and Bayesian correlation (bayesfactor package; Morey et al., 2015). In Bayesian correlation analysis, the null hypothesis H_0_ is *r* < 0.8, and the alternative hypothesis H_1_ is *r* $\ge$ 0.8. The Bayes factor for the alternative hypothesis, BF_10_, was calculated. As illustrated in Table 8, the test–retest correlations of three tasks between Time 1 and Time 2 were all lower than 0.8 under both ambiguity and conflictive uncertainty, with BF_10_ <0.001. These Bayes factors showed extreme evidence for the null hypothesis (*r < *0.8). These correlations were below the criteria of good test–retest reliability, failing to support Hypothesis 2.

**Table 8:** Test–retest correlations between different preference measures

	*r*	95% CI	*p* (*r* = 0)	BF_10_ (*r* $\ge$ 0.8)
CE – A	.56	[.46,.63]	<.001	<0.001
CE – C	.50	[.41,.59]	<.001	<0.001
MP – A	.20	[.08,.31]	<.001	<0.001
MP – C	.24	[.12,.35]	<.001	<0.001
FBC	.26	[.14,.37]	<.001	<0.001

CE = certainty equivalent; MP = matching probability; FBC = forced binary choice; A = ambiguity; C = conflictive uncertainty

To test whether the random error could be balanced by averaging scores for a sample, the agreement test was assessed by the Bayesian paired *t*-test. In this Bayesian paired *t*-test, the null hypothesis H_0_ is *d* = 0, and the alternative hypothesis H_1_ is *d* $\ne$ 0. It found that there was no significant difference in the average preference measured by the CE tasks between Time 1 (*M* = 28.72, *SD* = 18.47) and Time 2 (*M* = 29.06, *SD* = 16.55) under ambiguity, *t*(267) = −0.85, *p* =.396, BF_10_ = 0.098. The Bayes factors showed strong evidence for the null hypothesis (*d* = 0). However, there was a significant difference in the average preference measured by the CE tasks between Time 1 (*M* = 27.27, *SD* = 18.10) and Time 2 (*M* = 29.94, *SD* = 18.19) under conflictive uncertainty, *t*(267) = −3.06, *p* =.002, BF_10_ = 6.579. The Bayes factors showed moderate evidence for the alternative hypothesis (*d* $\ne$ 0).

There was no significant difference in the average preference measured by the MP tasks between Time 1 (*M* = 45.00%, *SD* = 9.39) and Time 2 (*M* = 44.26%, *SD* = 9.41) under ambiguity, *t*(267) = 3.35, *p* =.526, BF_10_ = 0.084. Similarly, there was no significant difference in the average preference measured by the MP tasks between Time 1 (*M* = 44.27%, *SD* = 9.10) and Time 2 (*M* = 44.25%, *SD* = 9.15) under conflictive uncertainty, *t*(267) = 0.27, *p* =.783, BF_10_ = 0.071. These Bayes factors showed strong evidence for the null hypothesis (*d* = 0). The difference in the scores from the FBC tasks was assessed by McNemar’s (1947) test, which is a paired test for binary variables. According to McNemar’s test, there was no significant difference in the proportion of participants preferring ambiguity in Time 2 compared to Time 1, χ^2^ (1, *N* = 268) = 1.44, *p* =.230.

These results indicate that the average scores in the MP task and FBC task show good agreement across time, suggesting that averaging scores across the sample could be an effective way to address the low test–retest reliability of the behavioral measures.

Turning to the correlation between the CE/MP tasks and risk preference tasks (see Table 9). Similar to Experiment 1, the correlations between the CE task and the risk preference tasks were significantly different from zero under both ambiguity, *r* =.32, BF_10_ > 100, and conflictive uncertainty, *r* =.31, BF_10_ > 100, whereas the correlations between the MP task and the risk preference tasks were not significant.

**Table 9:** Correlation between different ambiguity preference measures and the risk preference measure

	*r*	95% CI	*p* (*r* = 0)	BF_10_ (*r* $\ne$ 0)
CE – A	.32	[.22,.40]	<.001	> 100
CE – C	.31	[.21,.40]	<.001	> 100
MP – A	.11	[.02,.22]	.025	0.881
MP – C	.07	[−.04,.17]	.199	0.149

CE = certainty equivalent; MP = matching probability; FBC = forced binary choice; A = ambiguity; C = conflictive uncertainty

### Discussion

Experiment 2 found that the convergent validity among all three uncertainty preference measures was below the acceptable criteria (*r* > 0.5; Gregory, 2004). This could be due to the low test–retest reliability of these measures, as the test–retest correlations of these measures were also below the criteria for good reliability (*r* > 0.8; Mohajan, 2017). The findings indicate that there is still a large proportion of measurement error in the process of measurement.

## Experiment 3

As the low test–retest reliability could be generated by the large proportion of measurement error unable to be controlled in the one-off assessment, Experiment 3 increased the number of repetitions in each measurement session and averaged the responses from multiple repetitions to balance the random error. Meanwhile, Experiment 3 also explored the validity and reliability of these measures in capturing people’s ambiguity and conflictive uncertainty preference in medical scenarios (Berger et al., 2013). Because the US and UK medical systems are different (Fry, 2012), the sample for this experiment was restricted to the US population.

### Method

#### Participants and design

A total of 311 English speakers aged 18 or older from the USA were recruited via Prolific and participated in the study. Of these, 152 were randomly assigned to the gambling scenarios, with 48% of participants male, 50% female, and 2% non-binary genders. This sample size is sufficient to detect a significant medium correlation (*r* = 0.3) with a 0.05 significance level and 0.98 power. The participants included 24% African, 5% Asian, 65% Caucasian, and 6% people from other cultural backgrounds. Their mean age was 42.68 (*SD* = 15.13). Seventy percent of participants (*n* = 106) completed the second measurement session 9 days after the first wave of data collection.

The 159 remaining participants were assigned to the medical scenario. This sample size is sufficient to detect a significant medium correlation (*r* = 0.3) with a 0.05 significance level and 0.98 power. The participants included 53% male, 43% female, and 4% non-binary genders. There were 13% African participants, 5% Asian, 72% Caucasian, and 10% from other cultural backgrounds. Their mean age was 40.52 (*SD* = 12.82). Seventy-five percent of participants (*n* = 120) completed the second measurement session 9 days after the first wave of data collection.

Participants were recruited through the online platform Prolific. They were compensated at a fixed rate of £2 for their participation in a survey with no additional incentive, for which the median duration was expected to be 23 minutes. This experiment is a mixed-subject design. The scenario type (gambling vs. medical) was a between-subject variable. The uncertainty type and probability interval were within-subject variables. Participants were randomly assigned to the gambling or medical scenario, and completed all the decision-making tasks at the time they joined the study and 9 days later.

#### Materials

The descriptions of the forced binary choice tasks, certainty equivalent tasks, and matching probability tasks in the gambling scenario are the same as in Experiment 1. The only difference was that the probability intervals changed from fixed 30–70% intervals to intervals varied from 10–90% to 40–60% in 5% increments.

In the medical scenarios, participants were instructed to imagine themselves as hospital directors and assume responsibility for selecting a treatment plan for 100 patients who exhibited the same symptom in their hospital. The hospital introduced a supplementary task to aid them in making the decision. In this supplementary task, they were asked to choose from different treatment options with varying probabilities of success.

The descriptions of the forced binary choice, certainty equivalent, and matching probability tasks in the medical scenario were adapted to fit the context. For example, in the certainty equivalent task, the sure-win option referred to improving the condition of a subset of patients, whereas in the matching probability task, the options involved varying probabilities of the treatment improving the condition of all 100 patients.

#### Procedure

Participants were invited to take part in this survey and were randomly assigned to either the medical or gambling scenarios. Each scenario involved two measurement sessions, with the second session occurring 9 days after the first one. In each session, participants were initially provided with an introduction to the tasks’ options and completed practice questions for each task variant. Following this, they went through the formal tasks sequentially in a randomized order.

In each type of task, there were seven repetitions, each with varying probability intervals ranging from [10%, 90%] to [40%, 60%]. The order of the probability intervals was also randomized. Participants completed the demographic questions at the end of the first measurement session, and they were invited to complete the second session 9 days later. Payment was issued after each session.

Participants’ preferences in the CE and MP tasks were averaged across the repetitions with different probability intervals. The Cronbach’s alpha (Cronbach et al., 1972) of these preferences consistently measured around or above 0.8 (see Appendix D), indicating that participants demonstrated consistency in their responses to the repetitions with varying probability intervals. The binary preference from the FBC tasks was recoded (preferring ambiguity as 1 and preferring conflictive uncertainty as 0) and averaged across repetitions with different probability intervals.

### Results

The means and standard deviations of participants’ scores in the CE and MP tasks and the proportion of participants preferring ambiguity in the FBC tasks are summarized in Table 10.

**Table 10:** Descriptive information for three preference measures

		Ambiguity			Conflictive uncertainty		
Measure		Mean	Median	*SD*	Mean	Median	*SD*
Time 1							
Gambling	CE	33.69	35.35	19.49	36.13	37.50	19.35
	MP	45.29%	46.07%	10.79	44.94%	46.43%	10.51
	FBC	0.63	0.71	0.32			
Medical	CE	45.03	43.93	13.63	47.56	47.50	14.71
	MP	44.96%	45.00%	8.93	44.77%	45.00%	8.34
	FBC	0.61	0.71	0.36			
Time 2 (9 days later)							
Gambling	CE	38.12	38.21	17.86	38.79	38.57	18.72
	MP	45.83%	45.71%	10.07	45.39%	45.36%	10.02
	FBC	0.67	0.71	0.35			
Medical	CE	45.65	47.50	14.39	46.80	47.68	14.59
	MP	46.17%	46.06%	9.82	45.88%	46.79%	10.55
	FBC	0.73	0.86	0.31			

CE = certainty equivalent; MP = matching probability; FBC = forced binary choice

The convergent validity between different uncertainty preference measures was calculated using the same procedure as Experiment 1. Table 11 summarizes the convergent validity between different uncertainty preference tasks at two measurement times. Positive correlations were found among the FBC, CE, and MP tasks, varying from.09 to.67 across different measurement sessions and scenarios. The correlations showed an improvement compared to the results in the previous experiments. Most of the correlations were still below 0.5, although a few of the Bayes factors were smaller than 0.3, showing evidence preferring the null hypothesis. The results failed to support Hypothesis 1.

**Table 11:** Convergent validity between different preference measures using aggregated preferences

	Time 1				Time 2 (9 days later)					
	*r*	95% CI	*p* (*r* = 0)	BF_10_ (*r* $\ge$ 0.5)	*r*	95% CI		*p* (*r* = 0)	BF_10_ (*r* $\ge$ 0.5)	
Gambling										
CE vs. MP – A	.45	[.30,.59]	<.001	1.626	.43	[.26,.57]		<.001		1.013
CE vs. MP – C	.36	[.19,.51]	<.001	0.180	.46	[.30,.60]		<.001		1.675
FBC vs. CE	.18	[.00,.36]	.036	<0.001	.09	[−.09,.28]		.362		<0.001
FBC vs. MP	.28	[.10,.45]	.008	<0.001	.30	[.13,.46]		<.001		<0.001
Medical										
CE vs. MP – A	.58	[.45,.68]	<.001	37.037	.67		[.56,.76]	<.001		>100
CE vs. MP – C	.49	[.35,.61]	<.001	3.534	.63		[.52,.73]	<.001		>100
FBC vs. CE	.16	[.01,.33]	.041	<0.001	.20		[.03,.37]	.022		<0.001
FBC vs. MP	.17	[−.00,.34]	.238	<0.001	.36		[.19,.50]	<.001		<0.001

CE = certainty equivalent; MP = matching probability; FBC = forced binary choice;

A = ambiguity; C = conflictive uncertainty

A follow-up analysis that compared the different scenarios revealed that the convergent validity between the CE and MP tasks was significantly higher in the medical scenario than the gambling scenario at Time 2, under both ambiguity (*z* = −2.60, *p* =.005) and conflictive uncertainty (*z* = −1.807, *p* =.035). However, no significant differences in convergent validity were found across scenarios more generally.

As shown in Table 12, the test–retest correlations of all tasks were significantly different from zero across the two measurement times. The test–retest correlations for the CE tasks varied from.61 to.75 under different types of uncertainty and scenarios. The test–retest correlations for the MP tasks varied from.50 to.61, while the test–retest correlations for the FBC tasks varied from.31 to.33. However, almost all the correlations were below the criteria of good test–retest correlation (*r* > 0.8), failing to support Hypothesis 2. A follow-up analysis of the compared scenarios revealed no significant difference among these test–retest correlations between scenarios.

**Table 12:** Test–retest correlation between different preference measures using aggregated preferences

	Gambling				Medical			
	*r*	95% CI	*p* (*r* = 0)	BF_10_ (*r* $\ge$0.8)	*r*	95% CI	*p* (*r* = 0)	BF_10_ (*r* $\ge$0.8)
CE – A	.67	[.54,.76]	<.001	<0.001	.64	[.52,.73]	<.001	<0.001
CE – C	.75	[.65,.82]	<.001	0.630	.61	[.48,.71]	<.001	<0.001
MP – A	.54	[.38, 66]	<.001	<0.001	.55	[.42,.67]	<.001	<0.001
MP – C	.50	[.34,.63]	<.001	<0.001	.61	[.49,.71]	<.001	<0.001
FBC	.33	[.15,.49]	<.001	<0.001	.31	[.14,.47]	<.001	<0.001

CE = certainty equivalent; MP = matching probability; FBC = forced binary choice; A = ambiguity; C = conflictive uncertainty

Additionally, the agreement between scores was assessed by the paired *t*-test (see Table 13). Most of the *t*-tests showed moderate to anecdotal evidence for the null hypothesis, except for one of the FBC tasks in the medical scenario. For the FBC tasks in the medical scenario, participants were more likely to prefer conflictive uncertainty over ambiguity, in Time 2 compared to Time 1, BF_10_ = 4.567.

**Table 13:** Agreement of aggregated ambiguity preference between different measurement times

		*M*	*SD*	*t*	*p* (*d* = 0)	BF_10_ (*d* $\ne$ 0)
CE – A	Time 1	35.77	20.51	−1.52	.131	0.329
	Time 2	38.12	17.86			
CE – C	Time 1	36.95	19.03	−1.42	.160	0.283
	Time 2	38.79	18.72			
MP – A	Time 1	45.52%	10.09	−0.31	.757	0.113
	Time 2	45.83%	10.07			
MP – C	Time 1	44.98%	10.54	−0.41	.131	0.117
	Time 2	45.39%	10.02			
FBC	Time 1	0.63	0.34	−1.09	.280	0.159
	Time 2	0.67	0.35			
CE – A	Time 1	46.07	13.83	0.38	.708	0.109
	Time 2	45.65	14.39			
CE – C	Time 1	48.79	14.16	1.71	.090	0.415
	Time 2	46.80	14.59			
MP – A	Time 1	45.32%	8.82	−1.06	.293	0.175
	Time 2	46.17%	9.82			
MP – C	Time 1	45.92%	8.06	0.05	.962	0.101
	Time 2	45.88%	10.55			
FBC	Time 1	0.62	0.36	−3.09	.003	4.567
	Time 2	0.73	0.31			

CE = certainty equivalent; MP = matching probability; FBC = forced binary choice; A = ambiguity; C = conflictive uncertainty

### Discussion

Experiment 3 found that the convergent validity between the CE and MP tasks improved, although most still did not meet the acceptable criteria (*r* > 0.5; Gregory, 2004). The improvement in convergent validity could be attributed to the increased test–retest reliability in the MP tasks. This experiment suggests that averaging scores from different repetitions can address the low convergent validity and test–retest reliability between measures.

## General discussion

This study aimed to investigate the convergent validity and test–retest reliability of three behavioral measures of uncertainty preference, including the forced binary choice, certainty equivalent, and matching probability tasks. Experiments 1 and 2 found that the convergent validity of these three uncertainty preference measures fell below the acceptable criterion (*r* > 0.5; Gregory, 2004) in both the gain and loss domains. Experiment 2 showed that the test–retest reliability of these measures failed to meet the acceptable criterion for good reliability (*r* > 0.8; Mohajan, 2017). Experiment 3 found that the convergent validity between CE and MP tasks improved as the test–retest reliability of the MP tasks increased, although most of the convergent validity was still below the acceptable criterion (*r* > 0.5; Gregory, 2004).

In this study, convergent validity coefficients ranged from −0.06 to 0.24 under the one-off assessment condition, and from 0.09 to 0.67 under the repeated measurement condition. Test–retest reliability ranged from 0.20 to 0.56 under the one-off condition, and from 0.31 to 0.75 under the repeated measurement condition. These values fell in the same range as previous studies on convergent validity and test-retest reliability of risk and uncertainty preference measures (Beauchamp et al., 2017; Cavatorta & Schröder, 2019; Crosetto & Filippin, 2016; Frey et al., 2017; Galizzi & Miniaci, 2016; Grüner et al., 2023; Hey et al., 2009; Kimball et al., 2008; Xu et al., 2024), reinforcing long-standing concerns about the psychometric adequacy of behavioral preference measures. Importantly, these results stand in clear contrast to theoretical expectations, which assume that risk and uncertainty preferences are consistent and stable across tasks and over time. This discrepancy is further underscored by the Bayes factors, which provided strong evidence against the hypotheses of good convergent validity and test–retest reliability.

In addition to these investigations, this study explored two potential reasons for the low convergent validity among the three behavioral measures of uncertainty preference. Experiment 1 examined whether additional sources of systematic errors contributed to the low convergent validity. However, the partial correlation between CE and MP tasks showed no improvement after controlling for risk preference, indicating that unshared systematic errors may not be responsible for the previously observed low convergent validity between CE and MP tasks. Experiments 2 and 3 examined the impact of random error and low reliability on the convergent validity among these measures. The results showed that averaging preferences across trials or across individuals improved the reliability of individual scores and yielded strong consistency in average preferences over time. This suggests that the low reliability of these behavioral measures can be mitigated by aggregating scores across trials or individuals.

The findings in this paper should caution researchers about the potential misuse of the scores in the behavioral measures of risk and uncertainty preference as dependent variables when the validity and reliability of these measures are not well examined. Behavioral tasks have been widely applied to study people’s risk and uncertainty preferences, but their validity and reliability seem to be controversial given the present literature (Beauchamp et al., 2017; Coppola, 2014; Crosetto & Filippin, 2016; Frey et al., 2017; Hey et al., 2009). Researchers should be cautious about the selection of measures when studying people’s risk and uncertainty preference as a dependent variable, and may take actions (e.g., increasing the number of repetitions) to increase the validity and reliability of the measures when selecting a behavioral measure of risk and uncertainty preference.

Some limitations of this study should be noted. First, the ranges of outcomes and probabilities in this experiment were relatively limited. In the gambling scenarios, the outcomes ranged from $0 to $100, while in the medical context, the outcomes were about the conditions of 100 patients. Additionally, in both scenarios, the center of the probability interval was fixed at 50% in order to ensure a sufficient space of varying interval width. Participant responses may vary based on the magnitude of outcomes and the locations of probabilities (Kocher et al., 2018; Tversky & Kahneman, 1992). For example, participants’ attitudes towards ambiguity can become more positive when there is a low probability (e.g., 10%) of winning in a gambling game (Kocher et al., 2018). Additionally, the way probabilities are presented (e.g., as frequencies vs. ratios), along with how individuals interpret these probabilities, may influence the convergent validity and test–retest reliability of uncertainty preference measures. Therefore, future research should further investigate the validity and reliability of these measures across a broader range of scenarios, outcomes, and probability formats.

Second, this study primarily focused on the convergent validity and test–retest reliability of the uncertainty preference measures. It did not address other types of reliability and validity. Future research should explore the external and divergent validity of these measures, examining how preferences in these behavioral measures relate to real-world outcomes (Fairley & Weitzel, 2017; Frey et al., 2017).

Third, this study set 9 days as the interval between test–retest sessions, which may involve a memory effect. Although the memory effect is typically not considered a significant factor affecting test–retest reliability (McKelvie, 1992), future research could explore the reliability of ambiguity preference measures over longer time intervals.

In conclusion, this study explored the convergent validity and test–retest reliability of three uncertainty preference measures (forced binary choice task, certainty equivalent task, and matching probability task). It found that these measures showed unsatisfactory convergent validity and test–retest reliability in both one-off assessment and repeated measurement conditions. This highlights the measurement issues in risk and uncertainty preference studies. Researchers are encouraged to take further actions to enhance the validity and reliability of these measures before using their scores as a dependent variable in studies.

## Supplementary Information

Below is the link to the electronic supplementary material.

Supplementary file1 (DOCX 196 KB)

## Data Availability

The datasets collected during the current study are available from https://osf.io/nf5x9

## Code availability

The code used for analysis during the current study is available from https://osf.io/nf5x9
