# Supplementary material for: An investigation of the convergent validity and test–retest reliability of three uncertainty preference measures
Source: Behav Res Methods. 2025 Jul 9;57(8):221. doi: 10.3758/s13428-025-02729-9 (PMC12241273; doi:10.3758/s13428-025-02729-9)
Supplement: Supplementary file 1 — Supplementary file1 (DOCX 196 KB) [file 13428_2025_2729_MOESM1_ESM.docx]

**Appendix A**

**Figure A1**

*Example of a forced binary choice task*

**
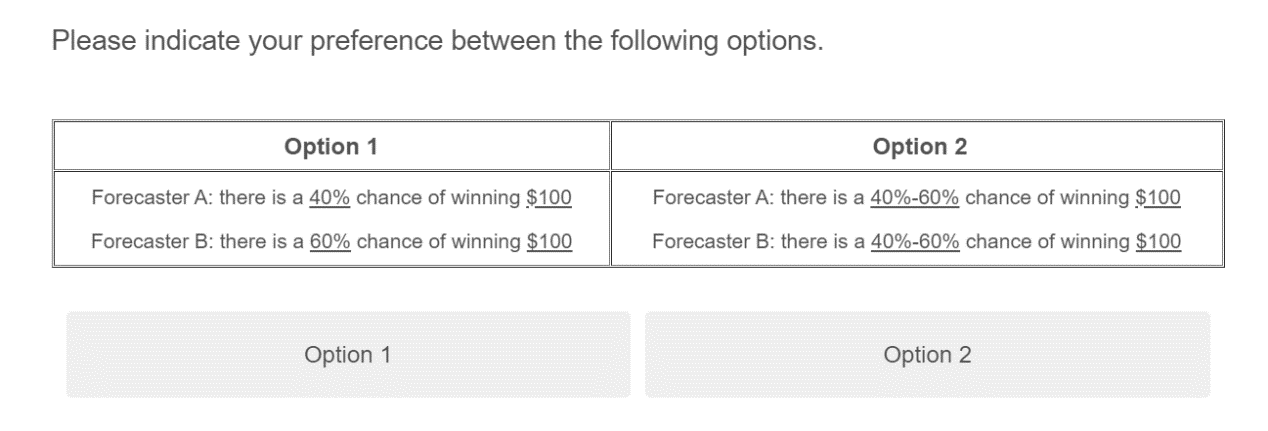
**

**Figure A2**

*Example of certainty equivalent task*


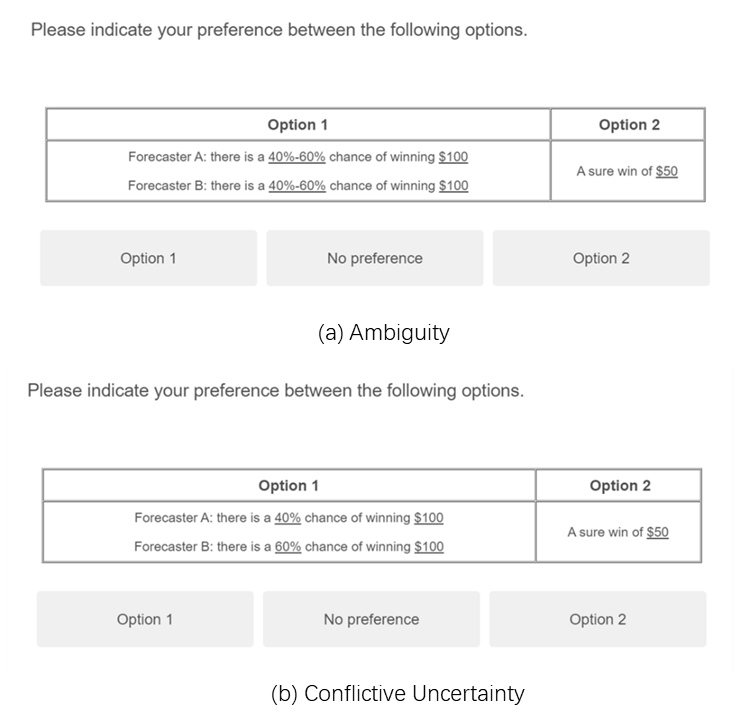


**Figure A3**

*Example of matching probability task*


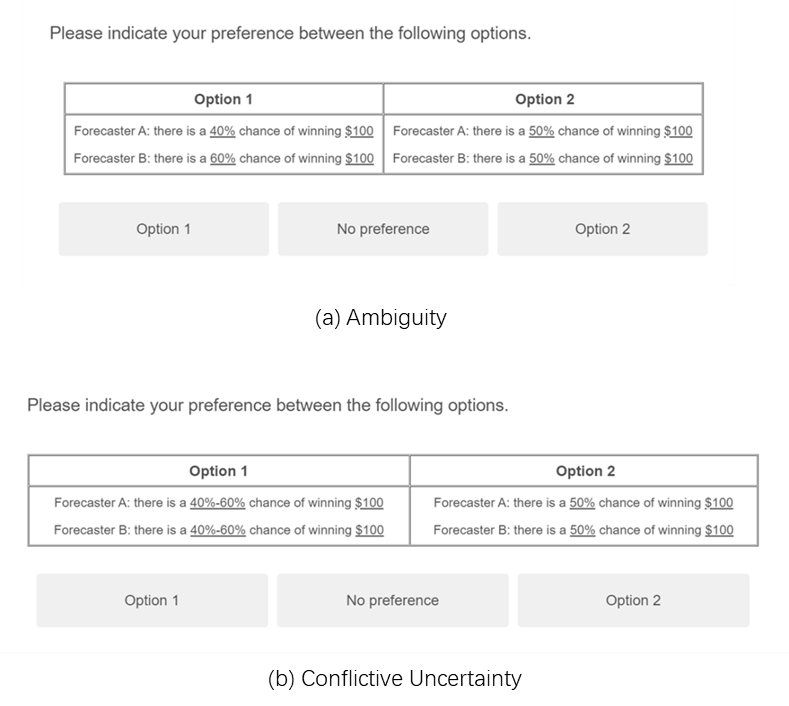


**Appendix B**

**Table B1**

*The payoff matrix in Holt and Laury (2002)*

| Lottery A | | | | Lottery B | | | |
| --- | --- | --- | --- | --- | --- | --- | --- |
| Payoff | p | payoff | p | payoff | p | payoff | p |
| $100 | 0.1 | $80 | 0.9 | $190 | 0.1 | $5 | 0.9 |
| $100 | 0.2 | $80 | 0.8 | $190 | 0.2 | $5 | 0.8 |
| $100 | 0.3 | $80 | 0.7 | $190 | 0.3 | $5 | 0.7 |
| $100 | 0.4 | $80 | 0.6 | $190 | 0.4 | $5 | 0.6 |
| $100 | 0.5 | $80 | 0.5 | $190 | 0.5 | $5 | 0.5 |
| $100 | 0.6 | $80 | 0.4 | $190 | 0.6 | $5 | 0.4 |
| $100 | 0.7 | $80 | 0.3 | $190 | 0.7 | $5 | 0.3 |
| $100 | 0.8 | $80 | 0.2 | $190 | 0.8 | $5 | 0.2 |
| $100 | 0.9 | $80 | 0.1 | $190 | 0.9 | $5 | 0.1 |
| $100 | 1 | $80 | 0 | $190 | 1 | $5 | 0 |

**Table B2**

*The payoff matrix in Chakravarty and Roy (2009)*

| Lottery A | | | | Lottery B | | | |
| --- | --- | --- | --- | --- | --- | --- | --- |
| payoff | p | payoff | p | payoff | p | payoff | p |
| -$40 | 0.5 | -$60 | 0.5 | $0 | 0.1 | -$100 | 0.9 |
| -$40 | 0.5 | -$60 | 0.5 | $0 | 0.2 | -$100 | 0.8 |
| -$40 | 0.5 | -$60 | 0.5 | $0 | 0.3 | -$100 | 0.7 |
| -$40 | 0.5 | -$60 | 0.5 | $0 | 0.4 | -$100 | 0.6 |
| -$40 | 0.5 | -$60 | 0.5 | $0 | 0.5 | -$100 | 0.5 |
| -$40 | 0.5 | -$60 | 0.5 | $0 | 0.6 | -$100 | 0.4 |
| -$40 | 0.5 | -$60 | 0.5 | $0 | 0.7 | -$100 | 0.3 |
| -$40 | 0.5 | -$60 | 0.5 | $0 | 0.8 | -$100 | 0.2 |
| -$40 | 0.5 | -$60 | 0.5 | $0 | 0.9 | -$100 | 0.1 |
| -$40 | 0.5 | -$60 | 0.5 | $0 | 1 | -$100 | 0 |

**Appendix C**

The Bayes factor compares the ratio of posterior probabilities of two hypotheses against the ratio of their prior probabilities:

$${BF}_{10}=\frac{PrPr (H_{1} | D)}{PrPr (H_{0} | D)}\times\frac{Pr(H_{0})}{Pr(H_{1})}$$

If *H*_0_ = $\rho\leq0.5$, *H*_1_ = $\rho> 0.5$, and $D = \{r, N\}$ where $\rho$ is the population correlation, $r$ is the sample correlation, and $N$ is the sample size, then our Bayes factor formula becomes

$${BF}_{10}=\frac{PrPr (\rho\geq0.5 | D)}{PrPr (\rho> 0.5 | D)}\times\frac{Pr(\rho< 0.5)}{Pr(\rho\geq0.5)}$$

The prior distribution for correlation $\rho$ is a uniform distribution from [-1, 1], which indicates that all values within this range have equal prior likelihood. By assuming a uniform distribution as the prior distribution for $\rho$, the prior odds:

$$\frac{Pr(\rho< 0.5)}{Pr(\rho\geq0.5)}=\frac{3}{1} = 3$$

The posterior odds $\frac{PrPr (\rho\geq0.5 | D)}{PrPr (\rho< 0.5 | D)}$ can be estimated from the simulated posterior samples (bayesfactor package: Morey et al., 2015). Thus, the Bayes factor can be calculated from the data.

One might argue that employing the uniform distribution as the prior could introduce a bias in the Bayes factor, favoring the alternative hypothesis, especially since the prior odds are already larger than 3. However, it is important to note that the posterior odds in most cases examined in this paper is much less than 0.001. As a result, the impact of the prior odds on the Bayes factor is minimal.

**Appendix D**

**Table E1**

*Cronbach’s alpha for participants’ responses in certainty equivalent and matching probability tasks*

|  | Gambling  (n = 152; Time 1) | Gambling  (n = 106; Time 2) | Medical  (n = 159; Time 1) | | Medical  (n = 120; Time 2) |
| --- | --- | --- | --- | --- | --- |
| CE – A | .96 | .95 | .93 | .96 | |
| CE – C | .95 | .96 | .93 | .95 | |
| MP – A | .86 | .89 | .85 | .88 | |
| MP – C | .85 | .86 | .82 | .90 | |
| FBC | .79 | .74 | .85 | .83 | |

*Note*. CE = Certainty equivalent; MP = Matching probability; FBC = Forced binary choice; A = Ambiguity; C = Conflictive uncertainty.
